# *In Vitro* and *In Vivo* Antimicrobial Activity of Hypochlorous Acid against Drug-Resistant and Biofilm-Producing Strains

**DOI:** 10.1128/spectrum.02365-22

**Published:** 2022-10-03

**Authors:** Marta Palau, Estela Muñoz, Enric Lujan, Nieves Larrosa, Xavier Gomis, Ester Márquez, Oscar Len, Benito Almirante, Jordi Abellà, Sergi Colominas, Joan Gavaldà

**Affiliations:** a Antibiotic Resistance Laboratory, Vall d’Hebron Research Institute (VHIR), Infectious Diseases Department, Vall d’Hebron University Hospital, Barcelona, Spain; b Spanish Network for Research in Infectious Diseases (REIPI RD19/0016), Instituto de Salud Carlos III, Madrid, Spain; c CIBERINFEC, ISCIII—CIBER de Enfermedades Infecciosas, Instituto de Salud Carlos III, Madrid, Spain; d Electrochemical Methods Laboratory-Analytical and Applied Chemistry Department, IQS School of Engineering, Universitat Ramon Llull, Barcelona, Spain; e Microbiology Department, Vall d’Hebron University Hospital, Barcelona, Spain; f Infectious Diseases Department, Vall d’Hebron University Hospital, Barcelona, Spain; INTHERES

**Keywords:** *A. fumigatus*, antibiotic lock technique, *Candida* spp., catheter-related infection, HClO, MDR bacteria, XDR bacteria, biofilms

## Abstract

The aims of this study were as follows. First, we determined the antimicrobial efficacy of hypochlorous acid (HClO) against bacterial, fungal, and yeast strains growing planktonically and growing in biofilms. Second, we sought to compare the activity of the combination of daptomycin and HClO versus those of the antimicrobial agents alone for the treatment of experimental catheter-related Staphylococcus epidermidis infection (CRI) using the antibiotic lock technique (ALT) in a rabbit model. HClO was generated through direct electric current (DC) shots at determined amperages and times. For planktonic susceptibility studies, 1 to 3 DC shots of 2, 5, and 10 mA from 0 to 300 s were applied. A DC shot of 20 mA from 0 to 20 min was applied to biofilm-producing strains. Central venous catheters were inserted into New Zealand White rabbits, inoculated with an S. epidermidis strain, and treated with saline solution or ALT using daptomycin (50 mg/mL), HClO (20 mA for 45 min), or daptomycin plus HClO. One hundred percent of the planktonic bacterial, fungal, and yeast strains were killed by applying one DC shot of 2, 5, and 10 mA, respectively. One DC shot of 20 mA for 20 min was sufficient to eradicate 100% of the tested biofilm-producing strains. Daptomycin plus HClO lock therapy showed the highest activity for experimental CRI with S. epidermidis. HClO could be an effective strategy for treating infections caused by extensively drug-resistant or multidrug-resistant and biofilm-producing strains in medical devices and chronic wounds. The results of the ALT using daptomycin plus HClO may be promising.

**IMPORTANCE** Currently, drug-resistant infections are increasing and there are fewer antibiotics available to treat them. Therefore, there is an urgent need to find new antibiotics and nonantimicrobial strategies to treat these infections. We present a new nonantibiotic strategy based on hypochlorous acid generation to treat long-term catheter-related and chronic wounds infections.

## INTRODUCTION

Biofilm-producing microorganisms are considered medically crucial, as they are the cause of 80% of the infections that occur in our body, according to an announcement by the U.S. National Institutes of Health (1). Some pathogens have the ability to grow as a community of microorganisms enclosed by a self-produced extracellular polymeric matrix and to attach to a wide variety of biotic and abiotic surfaces, such as medical devices or wounds, causing chronic infections (2, 3). This aggregate is known as biofilm; it has low growth rates and acts as a diffusion barrier for antimicrobial agents (4, 5). These resistance mechanisms contribute to a 10-1000-fold reduction in susceptibility against antimicrobial agents in comparison with their planktonic counterparts, causing difficult-to-treat infections (1, 4, 5). Likewise, another challenge is the enhanced resistance of the cells forming part of the biofilm caused by the efficient horizontal transfer and uptake of resistance genes (5, 6).

Every year, 700,000 people die due to antimicrobial resistance (AMR) and 25,000 of them are European (7, 8), with an estimated cost of 9 billion euros per year in Europe (8). Furthermore, if the situation is not reversed, it is estimated that from today until 2050, this figure will increase to 10 million deaths globally per year (7, 9, 10).

Common antimicrobial treatments typically fail to eradicate biofilm-related infections, necessitating surgical removal of the infected device (1). Therefore, device-associated infections represent a serious challenge in medical health care (1), such as long-term catheter-related infections (CRI), which can lead to bacteremia. Coagulase-negative staphylococci, which include Staphylococcus epidermidis, are considered the leading cause of CRI (50 to 70% of the cases) (11, 12) and are responsible for ~30% of the health care-associated bloodstream infections (BSIs). The recommended treatment in cases of uncomplicated long-term catheter-related BSIs is the antibiotic lock technique (ALT) using vancomycin (13). Unfortunately, this treatment fails in the form of relapse in 20% of episodes and lasts 14 days, resulting in reduced access to the catheter or even some patients having no other available vascular access (14).

Therefore, there is an urgent clinical need to find a faster and more effective treatment that sterilizes catheters, since the loss of an access vein or the replacement of the catheter due to infection can worsen the patient outcome (14). Moreover, the burden, morbidity, and health care costs of complicated chronic wounds and surgical site infections are currently increasing due to the increasing resistance of microorganisms to existing treatment alternatives (3, 15, 16).

In light of this critical scenario, there is an urgent need for new strategies with new mechanisms of action to tackle AMR and biofilm-related infections (17). The aim of this study was to treat infections caused by extensively drug-resistant (XDR) or multidrug-resistant (MDR) microorganisms and those growing on biofilms. The key is to mimic a reaction that takes place in nature, specifically inside the neutrophils, to combat the invading microorganisms, by inducing electrolysis of physiological saline (18). Despite the differences between the reaction mechanism of neutrophils and electrolysis, the final product is the generation of an antimicrobial nonantibiotic molecule, biologically classified as an oxygen reactive species and called hypochlorous acid (HClO) (19). It is extremely effective and fast and acts locally (20). It reacts with different molecules such as lipids, nucleotides, and proteins of microorganisms (20). Although its mechanism of action against microorganisms remains poorly understood, it seems to be a combination of different factors: inducing protein aggregation, decreasing cellular ATP, and inactivating essential bacterial chaperones that microorganisms excrete in response to stress (20).

Although some studies have reported the potential antimicrobial activity of HClO against a wide range of bacterial, fungal, and yeast strains, most of them have focused on using it only as a surface disinfectant or wound care agent (3, 5, 21, 22). Therefore, after confirming the high potential effect of HClO against clinical XDR/MDR Gram-negative and Gram-positive bacteria, *Candida* strains, and Aspergillus fumigatus strains, we present a new alternative: the use of HClO lock therapy for treating medical device-associated and wound infections caused by XDR/MDR and biofilm-producing strains.

This study had three aims. First, we evaluated the efficacy of HClO against the XDR/MDR bacterial, fungal, and yeast strains. Second, we assessed the antimicrobial activity of HClO against biofilm-producing strains growing on the surfaces of discs made of silicone, the material used in medical devices such as catheters, and in chronic wounds. Third, we evaluated the effectiveness of HClO for the treatment of experimentally induced S. epidermidis CRI by the ALT.

## RESULTS

### *In vitro* studies.

**(i) Quantification of the [HClO] generated.** The quantification of the [HClO] generated by applying 2, 5, 10, and 20 mA as a function of time is summarized in Tables 1 and 2.

**TABLE 1:** Quantification of the HClO concentration generated using *N*,*N*-diethyl-*p*-phenylenediamine sulfate colorimetric method by applying one, two, and three DC shots of 2 mA, 5 mA, and 10 mA from 20 to 300 s^*a*^

Time (s)	[HClO] (mM)								
	2 mA			5 mA			10 mA		
	1 DC	2 DC	3 DC	1 DC	2 DC	3 DC	1 DC	2 DC	3 DC
20	<0.002	<0.002	<0.002	<0.002	<0.002	<0.002	0.021	0.028	0.043
40	<0.002	<0.002	<0.002	<0.002	<0.002	0.007	0.045	0.085	0.139
60	<0.002	<0.002	0.002	<0.002	0.013	0.023	0.064	0.158	0.224
120	0.008	0.012	0.017	0.050	0.128	0.195	0.204	0.374	0.538
180	0.019	0.035	0.059	0.093	0.272	0.348	0.328	0.554	0.862
240	0.028	0.064	0.105	0.148	0.414	0.588	0.442	0.807	1.137
300	0.033	0.083	0.136	0.239	0.559	0.807	0.561	1.045	1.425

a DC, direct electric current; HClO, hypochlorous acid.

**TABLE 2:** Quantification of the HClO concentration generated using the *N*,*N*-diethyl-*p*-phenylenediamine sulfate colorimetric method by applying one DC shot of 20 mA from 5 to 45 min^*a*^

Time (min)	[HClO] (mM) with 1 DC of 20 mA
5	2.87
10	5.31
15	6.92
20	8.84
45	12.61

a DC, direct electric current; HClO, hypochlorous acid.

As shown in Tables 1 and 2, HClO generation increased proportionally to the electrochemical charge and number of direct electric current (DC) shots. Under conditions of low electrochemical charge, the measured HClO content was below the limit of quantification (0.002 mM).

**FIG 1:**
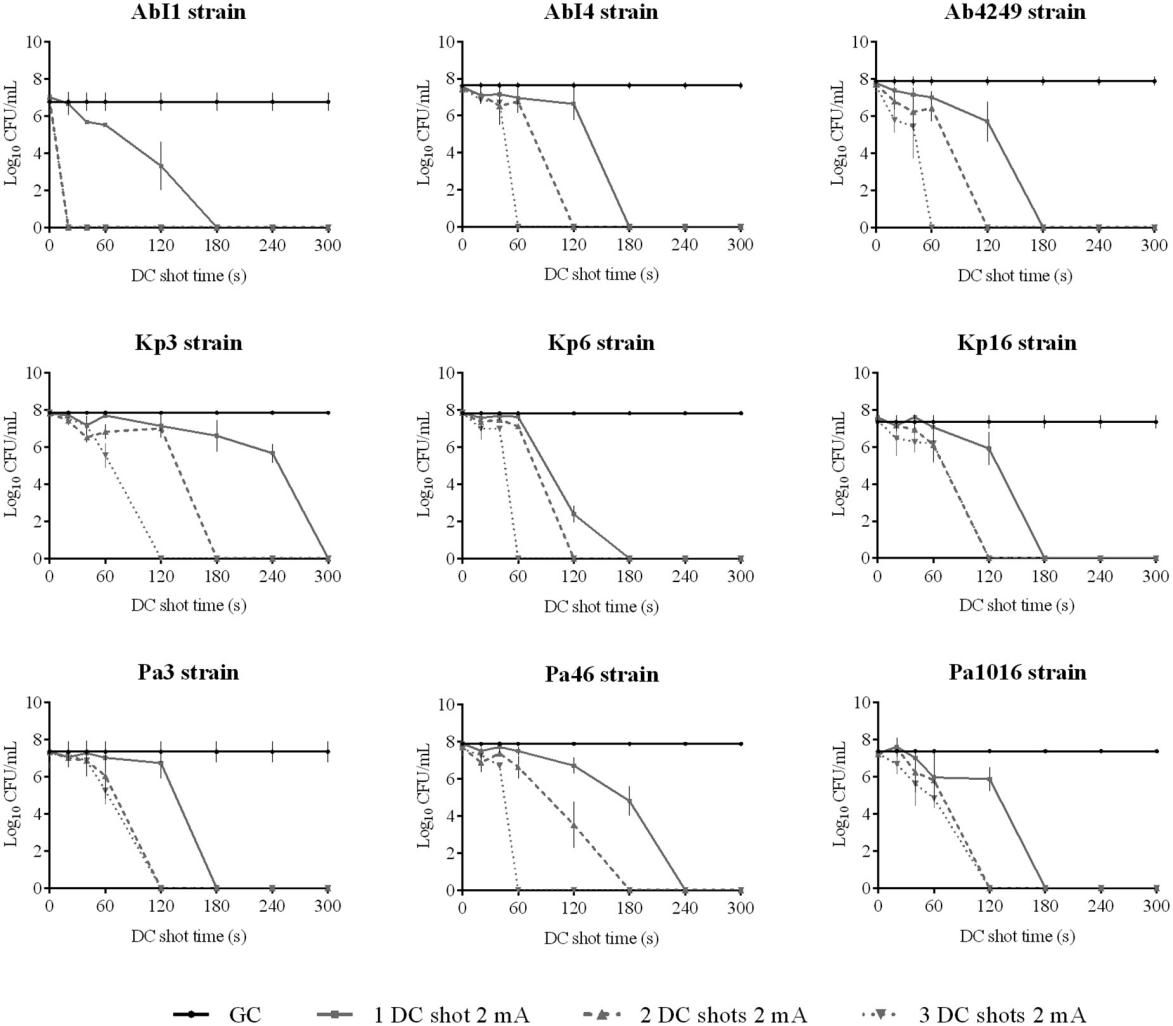
Efficacy of HClO by applying one, two, and three DC shots of 2 mA from 0 to 300 s against planktonically growing XDR/MDR Gram-negative clinical strains AbI1, AbI4, Ab4249, Kp3, Kp6, Kp16, Pa3, Pa46, and Pa1016. GC, growth control; DC, direct electric current; CFU, colony-forming units.

**FIG 2:**
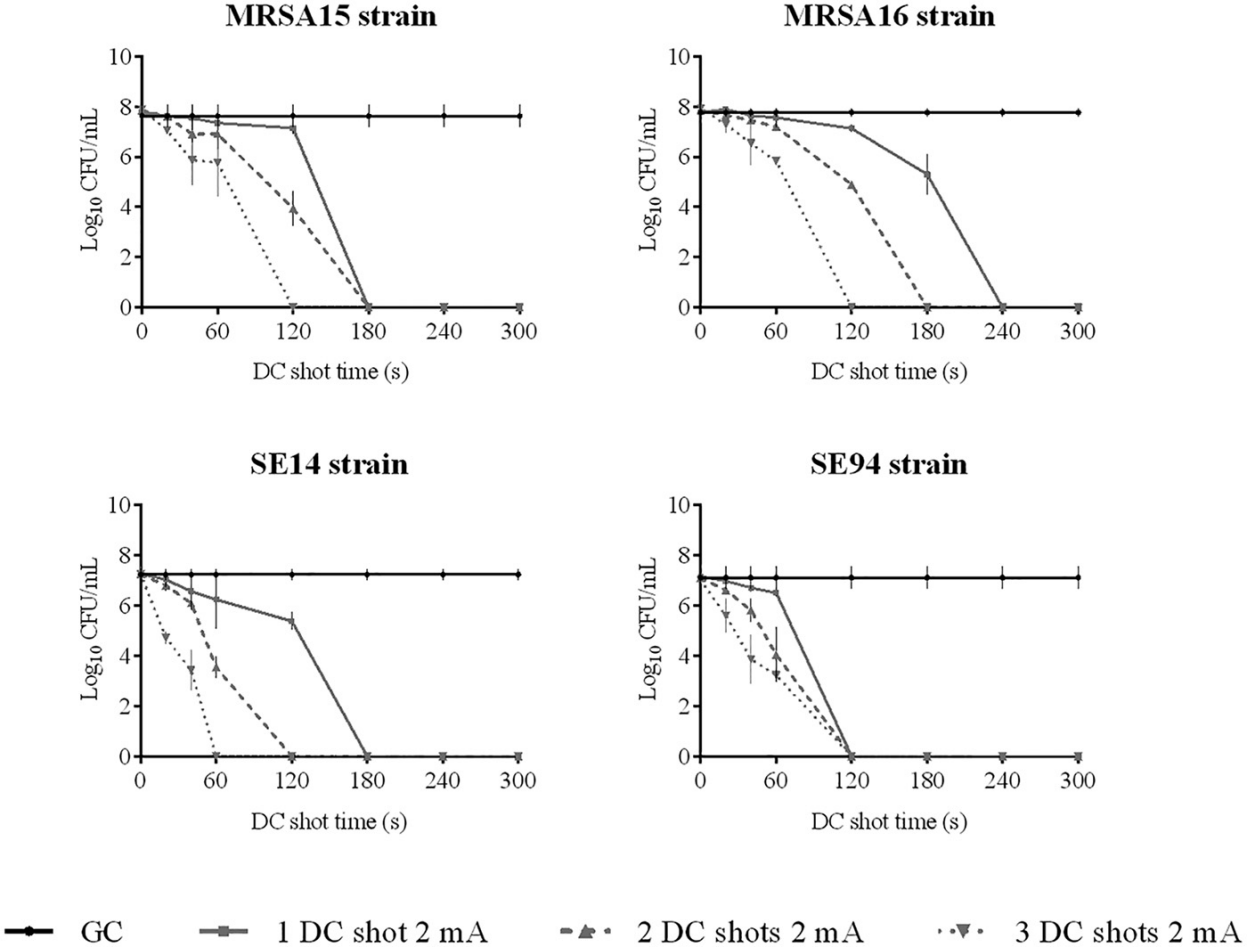
Efficacy of HClO by applying one, two, and three DC shots of 2 mA from 0 to 300 s against planktonically growing Gram-positive clinical strains MRSA15, MRSA16, SE14, and SE94. DC, direct electric current; CFU, colony-forming units; GC, growth control.

**FIG 3:**
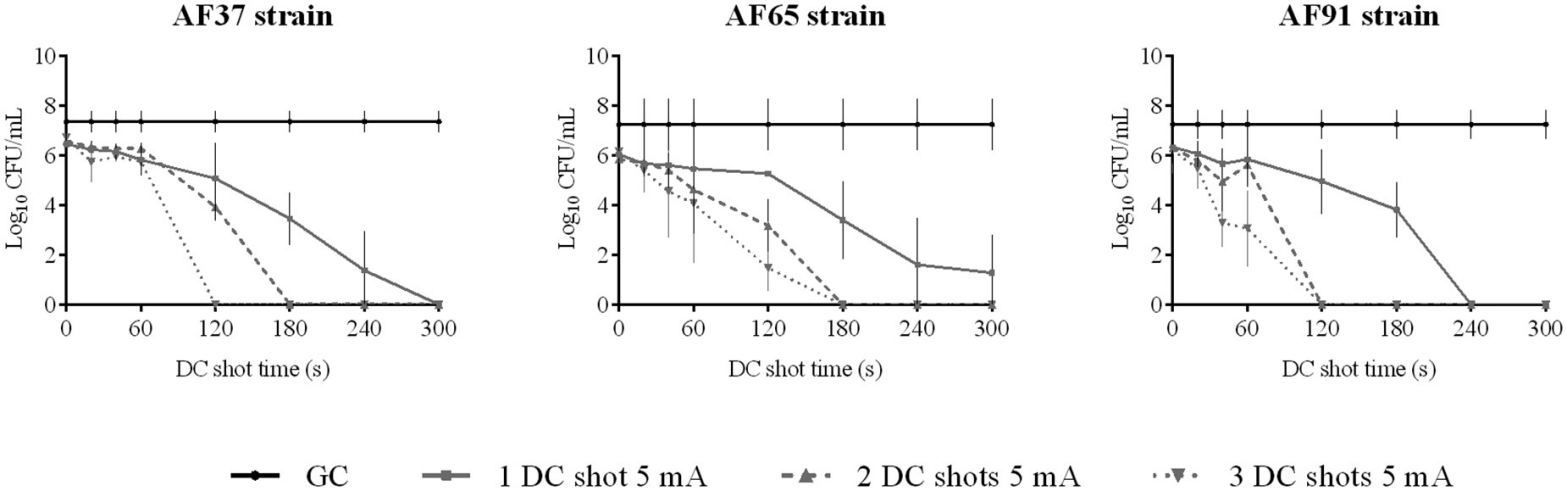
Efficacy of HClO by applying one, two, and three DC shots of 5 mA from 0 to 300 s against planktonically growing A. fumigatus clinical strains AF37, AF65, and AF91. DC, direct electric current; CFU, colony-forming units; GC, growth control.

**FIG 4:**
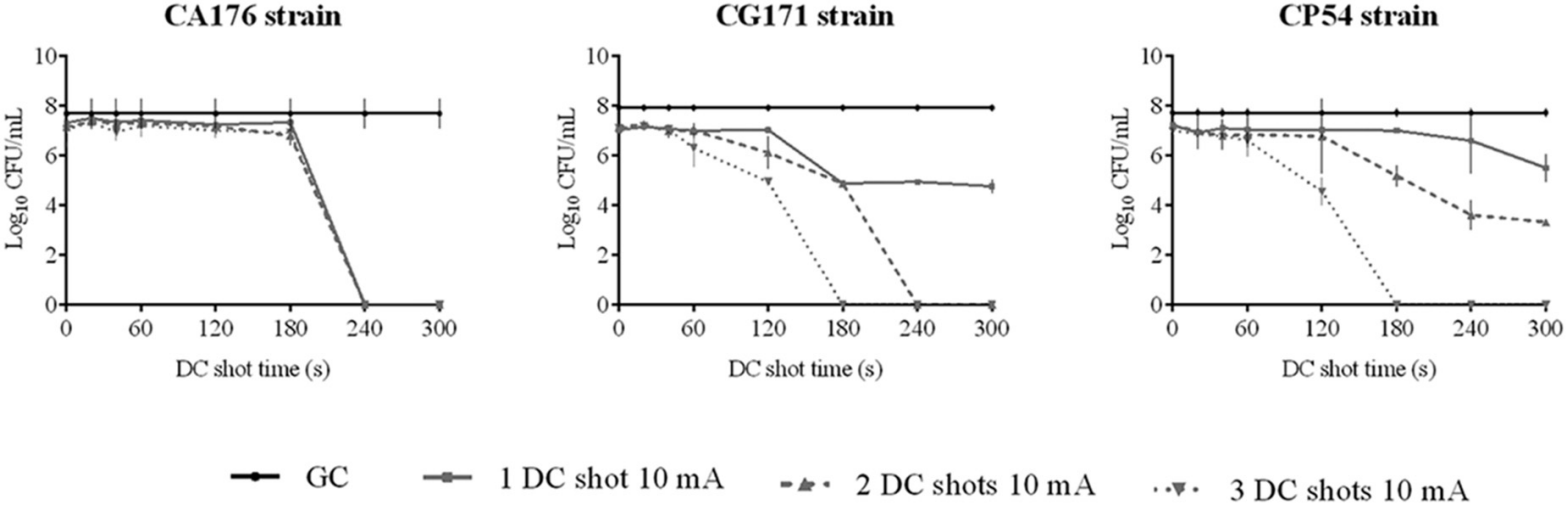
Efficacy of HClO by applying one, two, and three DC shots of 10 mA from 0 to 300 s against planktonically growing *Candida* clinical strains CA176, CG171, and CP54. DC, direct electric current; CFU, colony-forming units; GC, growth control.

**(ii) HClO efficacy assay against planktonically growing strains.** The efficacy of HClO against planktonically growing strains is shown in Fig. 1 to 4.

All the tested bacterial strains growing as planktonic cells were eradicated by applying a DC shot of 2 mA. Approximately three-fourths of the tested strains (10 out of 13 [77%]; AbI1, AbI4, Ab4249, Kp6, Kp16, Pa3, Pa1016, MRSA15, SE14, and SE94) were eradicated by applying only a DC shot of 2 mA for 3 min (equivalent to an [HClO] of 0.019 mM). A DC shot of 2 mA for 4 min (equivalent to an [HClO] of 0.028 mM) was necessary to eliminate Pa46 and MRSA16. Finally, Kp3 was killed by applying a DC shot of 2 mA for 5 min (equivalent to an [HClO] of 0.033 mM).

The administration interval and amperage had to be modified upward to kill A. fumigatus and *Candida* strains because they were less susceptible to the effect of HClO. These variables are species dependent. Both AF91 and AF37 strains were killed by applying a DC shot of 5 mA for 4 and 5 min (equivalent to [HClO] of 0.148 and 0.239 mM, respectively), whereas AF65 required two DC shots of 5 mA for 3 min (equivalent to an [HClO] of 0.272 mM). In the case of *Candida* spp., CA176 was killed by applying a DC shot of 10 mA for 4 min (equivalent to an [HClO] of 0.442 mM), whereas two DC shots of 10 mA for 4 min (equivalent to an [HClO] of 0.807 mM) and three DC shots of 10 mA for 3 min (equivalent to an [HClO] of 0.862 mM) were required to kill CG171 and CP54, respectively.

**(iii) HClO efficacy assay against biofilm-producing strains.** The efficacy of HClO against biofilm-producing strains growing on silicone discs was first determined using quantitative culture. Next, their morphology and viability were visualized and quantified using confocal laser scanning microscopy (CLSM) and LIVE/DEAD staining.

**FIG 5:**
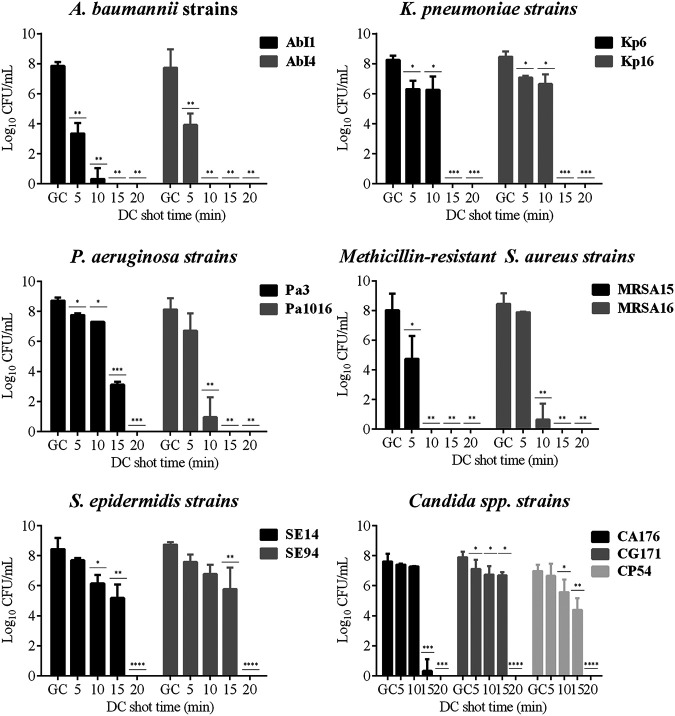
Efficacy of HClO by applying a DC shot of 20 mA from 0 to 20 min against biofilm-producing strains growing on silicone discs: A. baumannii (AbI1 and AbI4), K. pneumoniae (Kp6 and Kp16), P. aeruginosa (Pa3 and Pa1016), methicillin-resistant S. aureus (MRSA15 and MRSA16), S. epidermidis (SE14 and SE94), and *Candida* spp. (CA176, CG171, and CP54), obtained by quantitative culture. *, *P* ≤ 0.05 versus control; **, *P* ≤ 0.05 versus control and HClO at 20 mA for 5 min; ***, *P* ≤ 0.05 versus control, HClO at 20 mA for 5 min, and HClO at 20 mA for 10 min; ****, *P* ≤ 0.05 versus control, HClO at 20 mA for 5 min, HClO at 20 mA for 10 min, and HClO at 20 mA for 15 min. DC, direct electric current; GC, growth control.

**(iv) Quantitative culture assay.** The HClO efficacy results obtained by the quantitative culture are shown in Fig. 5. All studied strains were killed by applying a DC shot of 20 mA for 20 min. Specifically, a DC shot of 20 mA for 10 min (equivalent to an [HClO] of 5.31 mM) was required to kill AbI4 and MRSA15, which were the most susceptible, whereas a DC shot of 20 mA for 15 min (equivalent to an [HClO] of 6.92 mM) was needed to eradicate AbI1, Kp6, Kp16, Pa1016, and MRSA16. Finally, a DC shot of 20 mA for 20 min (equivalent to an [HClO] of 8.84 mM) was required to kill Pa3, SE14, and SE94, the less susceptible strains.

Regarding the three *Candida* strains studied (CA176, CG171, and CP54), a DC shot of 20 mA for 20 min was needed to completely eradicate the biofilm, showing a lower susceptibility to the antimicrobial activity of HClO, as has also been observed in planktonic susceptibility studies.

**(v) Confocal laser scanning microscopy assay.** CLSM and LIVE/DEAD staining were used to visualize the morphology and quantify cell viability after applying a DC shot of 20 mA for 5, 10, 15, or 20 min against all the tested strains (Acinetobacter baumannii, Klebsiella pneumoniae, Pseudomonas aeruginosa, methicillin-resistant Staphylococcus aureus, S. epidermidis, Candida albicans, Candida glabrata, and Candida parapsilosis).

**FIG 6:**
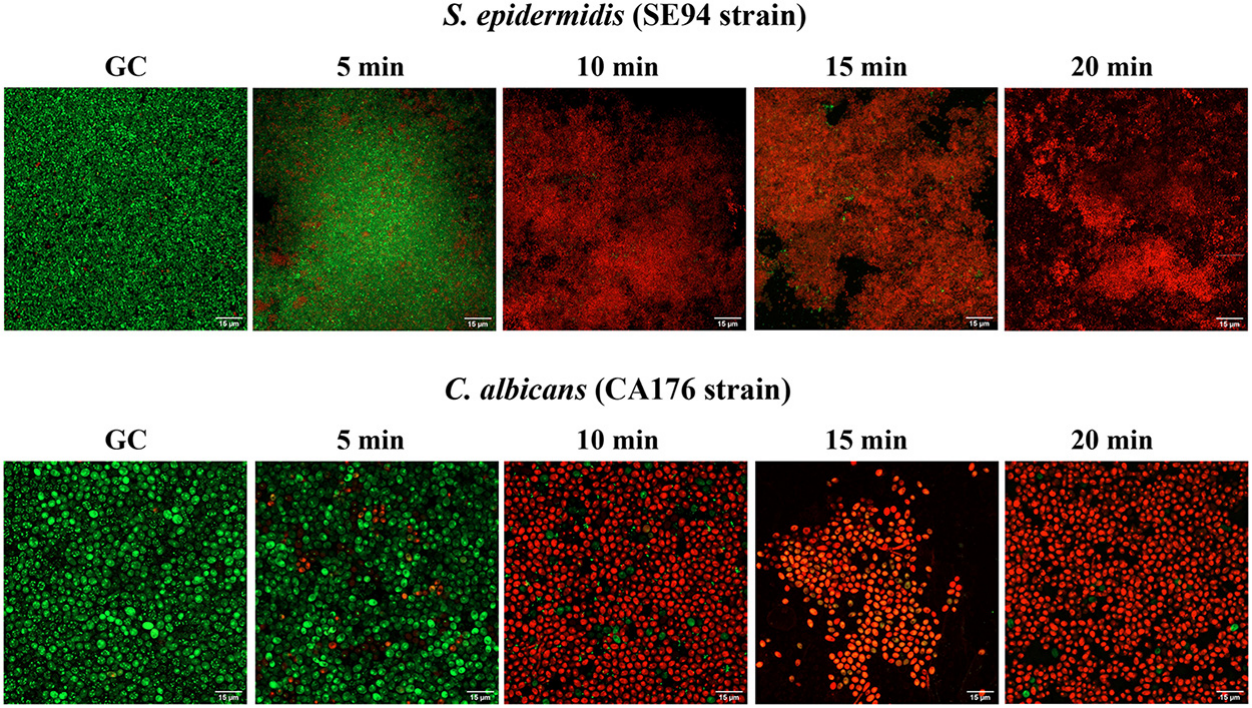
Two examples of the effect of HClO by applying a DC shot of 20 mA from 0 to 20 min on the morphology and cell viability of bacterial (SE94) and yeast (CA176) biofilm-producing strains growing on silicone discs. Images were obtained using confocal laser scanning microscopy and LIVE/DEAD staining. Red fluorescence, dead cells; green fluorescence, live cells (visualized at a magnification of ×60). GC, growth control.

As shown in Fig. 6, the application of HClO against the bacterial and yeast strains growing on silicone discs did not affect their morphology, as the shape and size of the treated cells did not change compared to those of the control group. Furthermore, LIVE/DEAD staining revealed the potential ability of HClO at high concentrations (8.84 mM) to diffuse down to the innermost layers of the biofilm in all the tested strains, as shown in Fig. 6. Although only two examples of the tested strains are shown in Fig. 6, the same results were obtained for all other clinical strains tested.

**FIG 7:**
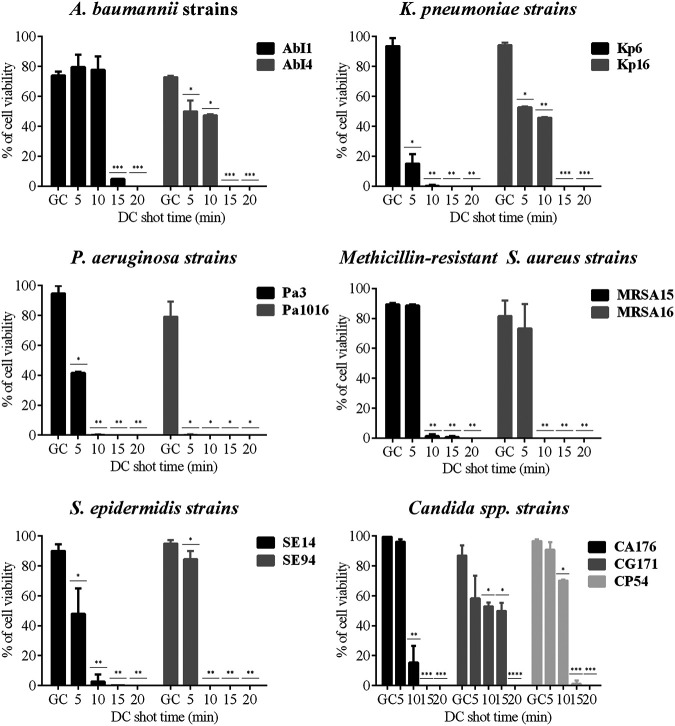
Efficacy of HClO by applying a DC shot of 20 mA from 0 to 20 min against different biofilm-producing strains growing on silicone discs: A. baumannii (AbI1 and AbI4), K. pneumoniae (Kp6 and Kp16), P. aeruginosa (Pa3 and Pa1016), methicillin-resistant S. aureus (MRSA15 and MRSA16), S. epidermidis (SE14 and SE94), and *Candida* spp. (CA176, CG171, and CP54), quantified from the images obtained by confocal laser scanning microscopy and LIVE/DEAD staining. *, *P* ≤ 0.05 versus control; **, *P* ≤ 0.05 versus control and HClO at 20 mA for 5 min; ***, *P* ≤ 0.05 versus control, HClO at 20 mA for 5 min, and HClO at 20 mA for 10 min; ****, *P* ≤ 0.05 versus control, HClO at 20 mA for 5 min, HClO at 20 mA for 10 min, and HClO at 20 mA for 15 min. DC, direct electric current; GC, growth control.

Moreover, the cell viability results (Fig. 7) were in agreement with the results obtained using the quantitative culture technique. All the biofilm-producing strains were killed by applying a maximum of one DC shot of 20 mA for 20 min (equivalent to an [HClO] of 8.84 mM). Specifically, half of the tested strains (Kp6, Pa3, Pa1016, MRSA15, MRSA16, and SE94) were more susceptible to HClO action, as only a DC shot of 20 mA for 5 or 10 min (equivalent to an [HClO] of 2.87 or 5.31 mM, respectively) was needed to kill them all. In contrast, 38% of the tested strains (AbI1, AbI4, Kp16, SE14, and CA176) were killed by applying a DC shot of 20 mA for 15 min (equivalent to an [HClO] of 6.92 mM). The remaining strains (CG171 and CP54) showed decreased susceptibility to HClO, as they were eradicated by applying a DC shot of 20 mA for 20 min (equivalent to an [HClO] of 8.84 mM).

### *In vivo* studies: catheter-related infection model.

The results of the ALT using HClO for one strain of S. epidermidis (SE14) in a CRI model are shown in Table 3.

**TABLE 3:** Results of antibiotic lock therapy for an S. epidermidis (SE14) strain in a rabbit catheter-related infection model^*a*^

Treatment	No. negative/total cultures (%)	Log_10_ CFU/mL, median ± SD (95% CI)
Control (SS, 0.9%)	0/11 (0)	6.00 ± 0.84 (5.01–6.14)
Daptomycin at 50 mg/mL	2/7 (29)	2.00 ± 1.74 (0.21–3.44)^*b*^
HClO (1 DC, 20 mA, 45 min)	0/5 (0)	4.25 ± 0.85 (2.89–5.01)^*b*^
Daptomycin at 50 mg/mL + HClO (1 DC, 20 mA, 45 min)	6/6 (100)^*b*^^,^^*c*^^,^^*d*^	0.00 ± 0.00 (0.00–0.00)^*b*^^,^^*c*^^,^^*d*^

a DC, direct electric current; SS, physiological saline solution; HClO, hypochlorous acid; CFU, colony-forming units; CI, confidence interval.

b *P* ≤ 0.05 versus control.

c *P* ≤ 0.05 versus daptomycin at 50 mg/mL.

d *P* ≤ 0.05 versus HClO (1 DC, 20 mA, 45 min).

Colony counts were significantly reduced (*P* ≤ 0.05), by 6 log_10_ colony-forming units (CFU)/mL, by treating the catheters with the combination of daptomycin at 50 mg/mL plus HClO (1 DC shot at 20 mA for 45 min) lock therapy (0.00 log_10_ CFU/mL) in comparison with the control group (6.00 log_10_ CFU/mL). Compared to the treatments alone (HClO, 4.25 log_10_ CFU/mL; daptomycin, 2.00 log_10_ CFU/mL), a significant reduction (*P* ≤ 0.05) was also achieved by treating the catheter with the combined lock therapy (0.00 log_10_ CFU/mL). Note that the combination of daptomycin plus HClO was the only therapy that achieved a significant percentage of sterilized catheters (100%) in comparison with the control group and the therapies alone (control group, 0%; daptomycin, 29%; HClO, 0%; *P* ≤ 0.05).

## DISCUSSION

The aims of the study were as follows. First, we determined the *in vitro* antimicrobial efficacy of HClO against XDR/MDR Gram-negative and Gram-positive bacterial, fungal, and yeast microorganisms growing as planktonic cells and growing in biofilms on the surfaces of silicone discs. Second, the *in vivo* effectiveness of the combination of daptomycin and HClO versus those of the antimicrobial agents alone for treating S. epidermidis CRI was assessed using the ALT.

In the *in vitro* planktonic study, we demonstrated that the generation of HClO has a rapid and powerful antimicrobial effect against nine XDR/MDR clinical isolates of A. baumannii, K. pneumoniae, and P. aeruginosa, two clinical isolates of methicillin-resistant S. aureus and S. epidermidis, three clinical isolates of *Candida* spp., and three strains of A. fumigatus. In addition, biofilm susceptibility studies against those strains growing on silicone discs have shown the ability of HClO at 8.84 mM to penetrate into the deepest layers of the biofilm, thereby completely eradicating it.

Focusing on planktonic assays, the applications of a DC shot of 2 mA for 5 min, two DC shots of 5 mA for 3 min, and three DC shots of 10 mA for 3 min were sufficient to kill 100% of the bacterial, fungal, and yeast strains, respectively. The efficacy of HClO against different bacterial, fungal, and yeast strains was also reported by Wang et al. (23), Sakarya et al. (21), and Shin et al. (22). Although they obtained successful efficacy results, the difference between the previously reported studies and our study is that while they tested the effect of HClO against a wide range of microorganisms, such as P. aeruginosa, S. aureus, S. epidermidis, K. pneumoniae, and even against yeasts and fungi such as C. albicans and Aspergillus niger, all of them were obtained from the American Type Culture Collection (ATCC), whereas we used clinical isolates obtained from patients at the Vall d’Hebron University Hospital. Our data also correlate with a study published by Herruzo and Herruzo (24), in which they studied the efficacy of HClO against P. aeruginosa, S. epidermidis, S. aureus, and C. albicans strains, all of which were isolated from the intensive care unit but were not XDR/MDR. They observed that 9.5 mM HClO was required to kill 1 × 10^6^ CFU/mL, whereas we used lower concentrations (8.84 mM) of the antimicrobial agent to kill higher bacterial, fungal, and yeast concentrations (1.5 × 10^8^ CFU/mL). However, the difference could be that they only allowed a wait time of 10 min maximum to observe the antibacterial effect of HClO after the exposure of the treatment, while we determined the HClO efficacy after 30 min.

Focusing on the susceptibility studies of biofilm-producing strains growing on silicone discs, we observed that all the tested strains were eradicated with only a DC shot of 20 mA for 20 min (8.84 mM HClO). The antibacterial effects of HClO against biofilm-producing strains were also reported by Chen et al. (25) and Kiamco et al. (3). Although the first study achieved results similar to ours by treating both Gram-positive and Gram-negative strains growing on titanium surfaces with HClO, it also demonstrated that the effect of HClO against Gram-negative bacteria could be explained by lipopolysaccharide removal from the cell membrane and, consequently, cytolysis induction (25). The second study required higher HClO concentrations (7 to 17 mM) and longer exposure times (1 to 3 h) than ours for treating P. aeruginosa, A. baumannii, and S. aureus infections in titanium scaffolds (3). A third study done by Flurin et al. (5) showed that a maximum HClO concentration of 29.50 mM in biofilm-producing monospecies caused a reduction in viable cells of >3 log_10_ CFU/cm^2^ after 4 h of exposure, whereas in our studies, we could completely eradicate the biofilm using a lower HClO concentration, 8.84 mA, during the 30 min of exposure in all the clinical strains tested. However, our results agree with the findings of Flurin et al. (5), as we observed that the antimicrobial effect of HClO is dose dependent, meaning that higher concentrations of the antimicrobial agent cause a greater reduction in bacterial viability.

These results also highlight the influence of the surface on biofilm formation and, therefore, the divergence in antimicrobial efficacy. As previously reported by our group (26), the maximum mimicry of infections that occur in clinics could be a key point in the study of antimicrobial therapies for treating CRI.

Moreover, our biofilm susceptibility studies on silicone discs were in agreement with those by Sandvik et al. (27). In their study, they applied DC for 24 h in the presence of saline solution (SS) against a biofilm-producing S. epidermidis strain grown in a CDC reactor. Considering both studies, we observed that the intensity of the DC and the electrolysis time increased the amount of HClO generation and, therefore, its antimicrobial activity. However, the main difference is that in our study, we only waited 30 min to observe the effect of the treatment, whereas in their study, they applied DC for a long period (24 h). In the future, if this technology is applied for medical purposes, it will be essential to reduce the time of exposure as much as possible for the patients’ comfort.

On the other hand, although our quantitative culture results mostly correlate with those obtained via CLSM and LIVE/DEAD staining techniques, some differences between the two techniques were observed. By comparing the techniques, it was determined that the quantitative culture assay showed that for some strains, higher concentrations of HClO were needed to eradicate the biofilm. The presence of extracellular DNA, incomplete replacement of SYTO 9 by propidium iodide (PI), energy transfer during costaining, and the viable-but-not-cultivable state of the cells are some of the factors that could explain these differences, resulting in an underestimation of cell viability (28–30). Despite the drawbacks of SYTO 9 and PI staining, the combination of this technique and quantitative culture can result in a more accurate estimation of cell viability (31–33).

The effect of pH on the generation of the HClO has been described previously (23, 25, 27). All studies agreed on the phenomenon of dissociation of HClO to the less antibacterial HClO^−^ form, which is highly dependent on pH. It has been reported that at pHs ranging between 6.3 and 6.7, 90% of the oxidation product is HClO, whereas with a pH increase, the concentration of HClO decreases and HClO^−^ becomes the most predominant form (3, 27). In our case, to maintain the pH at an approximate value of 6 and avoid obtaining undesired species, we used 100 mM phosphate-buffered saline (PBS; pH 6).

Infections associated with medical devices such as vascular catheters represent a major problem in medical practice owing to the formation of biofilms on their inner surfaces (34, 35). The current recommended solution for eradicating these associated infections is the removal of medical devices (26, 36). However, in certain clinical circumstances, such as in patients with hemorrhagic diathesis or absence of other venous access, this procedure is not always possible (26). Therefore, rapid sterilization of the catheter is essential to keep it in place. In our study, the *in vitro* biofilm susceptibility studies, providing a great model to mimic the biofilms that occur in clinics, showed that 20 mA for 20 min (8.84 mM) was sufficient to completely eradicate the biofilm-producing strains of A. baumannii, K. pneumoniae, P. aeruginosa, S. aureus, S. epidermidis, C. albicans, C. glabrata, and C. parapsilosis. As was also observed by Herruzo and Herruzo (24), if we compare the results of the biofilm-growing assay with the results obtained in the planktonic assay, higher amperage and longer exposition time of the treatment were required to kill biofilm-producing strains. This can be explained by the ability of biofilms to develop multiple mechanisms that allow them to evade antimicrobial agents and immune cells (4, 37). Moreover, our results showed that the exposure time of the treatment was species dependent. This was also reported by Kiamco et al. (3). Although HClO has broad-spectrum antimicrobial activity because it reacts with a number of cellular targets, the different exposure times between species could be explained by the different compositions and thicknesses of the membranes (3).

Antibiotics that treat infected catheters in patients are applied as an ALT, which consists of locking the lumen of the infected catheter with antibiotic solutions (38). Catheters infected with coagulase-negative staphylococci can be mainly sterilized with this technique and therefore kept in place and perform their function (38).

Daptomycin may be used as ALT for the treatment of coagulase-negative staphylococci-associated CRI. In a clinical case, Tatarelli et al. (39) studied the efficacy of daptomycin, with an average of 13 days (7 to 16 days duration) of use of the ALT (39). They concluded that daptomycin can be a good choice in patients in which the device cannot be removed thanks to its high-efficacy penetration into the biofilm (39).

In concordance with our studies, the ALT using daptomycin at a high dose (50 mg/mL) has been shown to have a higher activity against S. epidermidis CRI in a rabbit model by application of a 24-h treatment (40), in comparison with daptomycin at 5 mg/mL.

Some studies have shown that the combination of antibiotics in the ALT has a higher efficacy than treatments alone (41, 42). In agreement with that, our *in vivo* studies showed that the lock therapy using the combination of daptomycin at 50 mg/mL plus HClO achieved a significant reduction of the infection in comparison with that obtained with daptomycin alone. Furthermore, our study demonstrated that the lock therapy using this combination allowed a reduction in time to sterilize the catheter compared to that in a study done by Zhang et al. (42). In this study, the combination of vancomycin and ambroxol showed a significant reduction of the infection after 3 days of the ALT (42), while we had a total eradication of the infected catheters in only 5 h of the ALT. That difference can be translated to higher efficacy in clinical practice, including a reduction in the duration of treatment, which could be very beneficial in certain clinical circumstances.

Moreover, some antibiotics used in the ALT show a lack of activity against microorganisms at distant sites of the catheters (43). In contrast, our combination of daptomycin at 50 mg/mL plus HClO seems to reach the most distant part of the catheter, as our *in vivo* results showed a complete biofilm eradication in the distal part of the catheter.

In conclusion, our study demonstrated that HClO could be a promising strategy for treating difficult-to-treat infections caused by XDR/MDR and biofilm-producing bacterial, yeast, and fungal strains. Moreover, the results obtained by studying biofilm-producing strains growing on silicone discs could be a good approximation for further *in vivo* studies related to medical devices and wound infections. However, safety will need to be further assessed to confirm that HClO is harmless. Finally, the results obtained from the *in vivo* efficacy study of the ALT using HClO against an S. epidermidis CRI leads the strategy toward a faster and more efficient manner to sterilize infected catheters than the ones used in current clinical practice.

## MATERIALS AND METHODS

### Strains and resistance mechanisms.

For the *in vitro* planktonic susceptibility studies, nine clinical XDR/MDR Gram-negative isolates were studied: three strains of Acinetobacter baumannii (AbI1, AbI4, and Ab4249), three strains of Klebsiella pneumoniae (Kp3, Kp6, and Kp16) and three strains of Pseudomonas aeruginosa (Pa3, Pa46, and Pa1016). The sequence typing, the main acquired beta-lactam resistance mechanisms, and the results of antibiotic susceptibility testing (AST) of these Gram-negative strains are summarized in Table S1 in the supplemental material. Antimicrobial susceptibility was studied by the disc diffusion test (i2a, Montpellier, France) according to the European Committee on Antimicrobial Susceptibility Testing (EUCAST) guidelines (44). Additionally, susceptibility to antibiotics that are potentially active against some of these XDR/MDR isolates (ceftazidime, ceftazidime-avibactam, ceftolozane-tazobactam, piperacillin-tazobactam, cefiderocol, meropenem, meropenem-vaborbactam, imipenem-relebactam, amikacin, ciprofloxacin, trimethoprim-sulfamethoxazole, and colistin) was determined in duplicate by the microdilution technique using the Sensititre Gram-negative GN4F AST plate (Thermo Fisher Diagnostics S.L.U., Madrid, Spain). Discordance with the previous techniques was confirmed using a gradient test (Etest; bioMérieux SA, Marcy l’Etoile, France). P. aeruginosa ATCC 27853, Escherichia coli ATCC 25922, and K. pneumoniae ATCC 700603 and ATCC 2814 were used as quality control strains to determine the MIC.

Four Gram-positive clinical strains were also tested: two strains of methicillin-resistant Staphylococcus aureus (MRSA15 and MRSA16) and two strains of S. epidermidis (SE14 and SE94). Three clinical strains of fungi and yeast were also studied: three strains of A. fumigatus (AF37, AF65, and AF91) and one strain each of C. albicans (CA176), C. glabrata (CG171), and C. parapsilosis (CP54).

For the *in vitro* biofilm susceptibility studies on silicone discs, thirteen clinical strains were used: two A. baumannii (AbI1 and AbI4), two K. pneumoniae (Kp6 and Kp16), two P. aeruginosa (Pa3 and Pa1016), two methicillin-resistant S. aureus (MRSA15 and MRSA16), and two S. epidermidis (SE14 and SE94) strains and one strain each of C. albicans (CA176), C. glabrata (CG171), and C. parapsilosis (CP54).

For the *in vivo* susceptibility studies, one clinical Gram-positive strain of S. epidermidis (SE14) was studied.

All tested strains were isolated from patients at the Vall d’Hebron University Hospital.

All strains were stored in skim milk at −80°C in cryovial storage containers. Prior to each experiment, bacterial strains were subcultured in Trypticase soy agar (TSA; bioMérieux SA, Marcy l’Etoile, France) and incubated at 37°C for 24 h; fungal and yeast strains were subcultured on Sabouraud agar (Sigma-Aldrich Co., Madrid, Spain) plates with 8% glucose [d-(+)-glucose anhydrous; PanReac AppliChem, Barcelona, Spain] and were incubated at 37°C for 48 h.

### Substrates used in biofilm susceptibility studies.

For the *in vitro* biofilm susceptibility studies, strains were grown on the surfaces of silicone discs (diameter, 15 mm; thickness, 0.5 mm; Merefsa, Barcelona, Spain).

For the *in vivo* susceptibility studies, strains were grown in the lumen of silicone catheters (length, 18 cm; Ø_e_, 2 mm; Ø_i_, 1 mm; Merefsa, Barcelona, Spain).

### Antimicrobial agents.

**(i) HClO.** HClO was generated through direct electric current (DC) shots at determined amperages and times. DC was applied using two platinum wires (Sempsa JP, Madrid, Spain) as the working and counter electrodes. A potentiostat/galvanostat PalmSens4 (PalmSens, Houten, Netherlands) was used to perform all electrochemical measurements. One, two, or three DC shots of 2, 5, 10, and 20 mA were applied by immersing the platinum wires in 4 mL of a mixture of physiological saline solution (SS; Grifols, Barcelona, Spain) and 100 mM phosphate-buffered saline (pH 6; prepared by mixing disodium hydrogen phosphate dodecahydrate [Scharlab S.L., Barcelona, Spain] and sodium dihydrogen phosphate dihydrate [Scharlab S.L.] and adjusted with 5,000 mM sodium hydroxide [Panreac, Barcelona, Spain]).

Quantification of the [HClO] was performed using the *N*,*N*-diethyl-*p*-phenylenediamine sulfate (DPD; Merck KGaA, Darmstadt, Germany) colorimetric method (standard method 4500-Cl G), with some modifications (45).

First, DPD was prepared by mixing 150 mg of DPD, 20 mg of EDTA disodium salt (Panreac, Barcelona, Spain), and 1 mL of 1:3 sulfuric acid (Merck KGaA, Darmstadt, Germany) in up to 100 mL of deionized water. Subsequently, 300 μL of the DPD reactant, 300 μL of PBS (pH 6), and 100 μL of the sample were poured into a spectrophotometric cell. Absorbance was measured at 515 nm using a Perkin Elmer Lambda 25 UV-visible (UV-Vis) spectrophotometer (Perkin Elmer, MA, USA). Finally, to determine the [HClO], the measured absorbance was interpolated using a previously performed standard curve. The limit of quantification (LOQ), which is the lowest analyte concentration that can be quantitatively detected with accuracy and precision, was estimated to be 0.002 mM.

**(ii) Antibiotic.** Daptomycin was purchased from Novartis Europharm Limited Horsham (West Sussex, United Kingdom) and prepared according to the manufacturer’s instructions. It was supplemented with 100 IU/mL of sodium heparin (Laboratorios Farmacéuticos ROVI, S.A., Madrid, Spain).

### *In vitro* studies.

**(i) HClO efficacy assay against planktonically growing strains.** First, in the case of bacterial and *Candida* strains, 4 mL of an inoculum of 1.5 × 10^8^ CFU/mL prepared with SS and 100 mM PBS (pH 6) was placed in each well of a 12-well plate (Sarstedt AG & Co., Nümbrecht, Germany).

For the preparation of A. fumigatus (AF37, AF65, and AF91) conidial suspension, a subculture of the clinical isolate was scraped from Sabouraud plates with 10 mL of physiological saline with 0.025% Tween 20 (Sigma, Steinheim, Germany). The suspension was washed twice with PBS (pH 7.2; Merck, Darmstadt, Germany), and conidia were counted using a Fuchs-Rosenthal counting chamber (Brand, Wertheim, Germany) and adjusted with SS and 100 mM PBS (pH 6) to a final concentration of 1.5 × 10^8^ cells/mL. Then, 4 mL of the suspension was added to each well of a 12-well plate.

Before applying the DC, the 12-well plate with the inoculum was incubated at 37°C for 15 min. Then, one, two, and three DC shots of 2 mA (for bacterial strains) or 5 mA and 10 mA (for fungal and yeast strains, respectively) for 20, 40, 60, 120, 180, 240, and 300 s were applied. After every shot, the plate was incubated for 30 min at 37°C and 120 μL of inoculum from each condition was collected. Finally, efficacy of the HClO was determined by counting the number of CFU/mL at established times by plating them in TSA and incubating the plates at 37°C for 24 h (bacterial strains) or in Sabouraud agar plates and incubating them at 37°C for 48 h (fungal and yeast strains). All experiments were performed in triplicate.

**(ii) HClO efficacy assay against biofilm-producing strains.** For biofilm formation, the protocol described by Chandra et al. (46) was followed, with some modifications. The biofilm-producing strains were grown overnight in tryptic soy broth (TSB; Becton, Dickinson and Company, Le Pont Claix, France) for bacterial strains and in brain heart infusion medium (BHI; Sigma-Aldrich Co., Madrid, Spain) for yeast strains, and both were incubated at 37°C and 60 rpm. After centrifugation at 3,500 rpm for 5 min at 4°C and washing of the cell suspension three times with sterile PBS (pH 7.2), an inoculum of 1 × 10^7^ CFU/mL was prepared in PBS (pH 7.2). Next, 4 mL of inoculum and silicone discs were added to each well of a 12-well plate. The plate was incubated for 90 min at 37°C (adhesion step), and the discs were then placed in a new plate containing 4 mL of fresh TSB (bacterial strains) or BHI (yeast strains). The plate was incubated for 24 h (bacterial strains) or 48 h (yeast strains) at 37°C with stirring at 60 rpm (growth step).

To evaluate the efficacy of HClO, 4 mL of the mixture of SS and 100 mM PBS (pH 6) and the silicone discs with the biofilm formed were placed in a new plate. Subsequently, a DC shot of 20 mA was applied for 5, 10, 15, and 20 min.

The efficacy of HClO was first evaluated by quantitative culture. The discs were transferred to a new 12-well plate containing 1 mL of TSB (bacterial strains) or BHI (yeast strains). The biofilm grown on the surface of the silicone discs was scraped (cell scraper; Sarstedt AG & Co., Nümbrecht, Germany), plated on TSA (bacterial strains) or Sabouraud agar (yeast strains), and incubated at 37°C for 24 h (bacterial strains) or 48 h (yeast strains). Finally, cells were quantified and counts were expressed as log_10_ CFU per milliliter.

The effect of the treatment was also visualized using a Zeiss LSM980 confocal laser scanning microscope (CLSM) with excitation wavelengths of 488 and 568 nm 30 min after the DC shot. Biofilms were stained using a LIVE/DEAD BacLight viability kit (Molecular Probes, Invitrogen, Leiden, The Netherlands) following the manufacturer’s instructions, which consisted of staining the samples with 200 μL of a mixture of SYTO 9 (3.34 mM solution in dimethyl sulfoxide [DMSO]) and propidium iodide (20 mM solution in DMSO) and incubating them at room temperature in the dark for 30 min. The mixture was prepared with 3 μL of each dye in 1 mL of sterile distilled water. Three areas of the biofilm on each silicone disc were scanned with a 2-μm step size. Simultaneous dual-channel imaging was used to display green (live cells) and red (dead cells) fluorescence. IMARIS 8 software (Bitplane, Belfast, UK) was used to create a projection view of the biofilms, and the ImageJ 1.45s software package was used to calculate the percentage of live (green) versus dead (red) pixels. All experiments were performed in triplicate.

The results of the HClO efficacy assay against biofilm-producing strains were analyzed using one-way analysis of variance (ANOVA) and the Tukey’s *post hoc* test. Statistical analysis was performed using the Statistical Package for the Social Sciences (SPSS, Inc., Chicago, IL, USA). *P* values of ≤0.05 were considered statistically significant.

### *In vivo* studies.

**(i) Animals.** For the *in vivo* experiments, New Zealand White male rabbits (Granja Cunícola San Bernardo, Navarra, Spain) weighing 2.0 to 2.2 kg were used. Upon arrival, animals were housed in individual cages under a reversed 12-h/12-h light/dark cycle and provided water and food *ad libitum* throughout the experiment. The experimental protocol was approved by the Ethics Committee for Animal Experimentation of Vall d’Hebron Research Institute (registration number 59/20 CEEA) and the Ministry of Environment of the Catalan Government (registration number 11306).

**(ii) Animal model.** Rabbits were anesthetized by an intramuscular injection of 15 mg/kg of body weight of ketamine (Pfizer, Madrid, Spain) plus 0.25 mg/kg of medetomidine (B. Braun, Barcelona, Spain). When animals had no corneal or foot reflex, each rabbit’s neck region was cleaned with ethanol and povidone iodine prior to incision. A small skin incision (2 cm) was made under sterile conditions over the right paratracheal region to expose the jugular vein bifurcation. The external jugular vein was distally tied with USP 3/0 surgical silk suture (Silkam black; B. Braun, Barcelona, Spain) and a small venotomy was made through which about 8 cm of a sterile catheter of 18 cm was introduced (2 mm [Ø_e_] by 1 mm [Ø_i_]; Merefsa, Barcelona, Spain) into the superior cava vein. Silk sutures were used to fix the positioning of the catheter inside the superior cava vein. The incision was then closed with uninterrupted stitches. The catheter was then subcutaneously tunneled, brought to the interscapular region by making a small incision (0.5 cm), and fixed to the skin with stitches. The patency was checked by drawing blood and flushing with SS.

After removal from anesthesia, catheters were inoculated with 0.3 mL of S. epidermidis (SE14) suspension containing 1 × 10^8^ CFU/mL prepared with TSB plus 0.5% glucose. Twenty-four hours later, the inoculum was withdrawn by aspiration and 0.3 mL of SS was flushed. Forty-eight hours after surgery, catheters were again flushed with 0.3 mL of SS. All locks were supplemented with 100 IU of sodium heparin/mL. Animals’ health and absence of inflammation of the incisions were daily checked during the study.

**(iii) Treatment groups and therapeutic efficacy.** HClO at a concentration of 12.6 mM was generated *in vitro* by applying one DC shot of 20 mA for 45 min.

Seventy-two hours postinfection, the animals were randomly classified into the following groups: control SS locked for 5 h, 50 mg/kg of daptomycin for 3 h, 12.6 mM HClO for 2 h, and 50 mg/mL of daptomycin for 3 h plus 12.6 mM HClO for 2 h. The catheter was locked with 0.3 mL of the respective antimicrobial agent.

At the end of the treatment period, animals were anesthetized by an intramuscular injection of 15 mg/kg of ketamine plus 0.25 mg/kg of medetomidine. When animals had no corneal or foot reflex, a paratracheal incision was done and the catheter was removed under sterile conditions. A 4-cm piece of the distal part of the catheter was cut and flushed with TSB with 0.5% glucose to remove the remaining antimicrobial agent. The catheter was bisected lengthwise, placed in 6 mL of TSB plus 0.5% glucose, and sonicated at 50 Hz for 10 min. The pellet was washed twice by centrifugation at 3,500 rpm for 10 min, and the final pellet was diluted and plated in TSA for quantitative culture.

Results were expressed as log_10_ CFU per milliliter and analyzed by the nonparametric Mann-Whitney test. The percentages of negative cultures obtained were compared between treatments using Fisher’s exact test. Statistical analysis was performed using SPSS. *P* values of ≤0.05 were considered statistically significant.
