# Supplementary material for: In Vitro and In Vivo Antimicrobial Activity of Hypochlorous Acid against Drug-Resistant and Biofilm-Producing Strains
Source: Microbiol Spectr. 2022 Oct 3;10(5):e02365-22. doi: 10.1128/spectrum.02365-22 (PMC9602778; doi:10.1128/spectrum.02365-22)
Supplement: Supplemental File 1 — Supplemental material. Download spectrum.02365-22-s0001.pdf, file, 0.02 MB [file spectrum.02365-22-s0001.pdf]

Table S1 Characteristics of the Gram-negative isolates used in the study

			Microdilution technique MIC (mg/L)									Disk-diffusion Zone diameter (mm)		
	**ST**	**Third-generation cephalosporin and carbapenemase resistance mechanisms**	**CTZ**	**CTV**	**CTT**	**PIT**	**MER**	**MEV**	**IMR**	**CIP**	**COL**	**FDC**	**AMI**	**TRS**
**AbI1**	103	NDM-2, OXA-51	>32 ND	> 8/4 ND	> 8/4 ND	> 64/4 ND	>16 R	8/8 ND	>8/4 R	4 R	0.5 S	16.1 R	6 R	6 R
**AbI4**	2	OXA-51	>32 ND	> 8/4 ND	> 8/4 ND	> 64/4 ND	>16 R	>8/8 ND	>8/4 R	> 4 R	1 S	20.5 R	22 S	6 R
**Ab4249**	24	OXA-24, OXA-51	>32 ND	> 8/4 ND	> 8/4 ND	> 64/4 ND	>16 R	>8/8 ND	>8/4 R	> 4 R	0.5 S	18.7 R	18 S	6 R
**Kp3**	ND	KPC, CTX-M	>32 R	2/4 S	> 8/4 R	> 64/4 R	>16 R	0.023 S	0.25/4 S	4 R	0.5 S	22.1 S	14 R	6 R
**Kp6**	ND	IMP, CTX-M	>32 R	> 8/4 R	> 8/4 R	> 64/4 R	8 R	8/8 R	0.5/4 S	2 R	0.125 S	25.5 S	20 S	6 R
**Kp16**	ND	DHA	>32 R	0.5/2 S	2/4 S	> 64/4 R	0.25-0.5 S	0.05/8 S	2/4 S	0.5 I	0.5 S	23.2 S	26 S	30 S
**Pa3**	235	VIM-2	>32 R	> 8/4 R	> 8/4 R	> 64/4 R	>16 R	>8/8 R	>8/4 R	> 4 R	2 S	24.6 S	11 R	6 R
**Pa46**	111	VIM-2	>32 R	> 8/4 R	> 8/4 R	> 64/4 R	>16 R	>8/8 R	>8/4 R	> 4 R	1 S	23.3 S	12 R	6 R
**Pa1016**	175	-	32 R	2/4 S	2/4 S	> 64/4 R	16 R	>8/8 R	4S	> 4 R	4 S	25.6 S	24 S	6 R

ST, sequence typing; MIC, minimum inhibitory concentration; ESBL, extended spectrum beta-lactamase; CTZ, ceftazidime; CTV; ceftazidime/avibactam; CTT, ceftolozane/tazobactam; PIT, piperacillin/tazobactam; MER, meropenem; MEV, meropenem/vaborbactam; IMR, imipenem/relebactam; CIP, ciprofloxacin; COL, colistin; FDC, cefiderocol; AMI, amikacin; TRS, trimethoprim/sulfamethoxazole; ND, not determined; R, resistant; S, susceptible.
